# Single nucleotide polymorphism-based analysis of the genetic structure of Liangshan pig population

**DOI:** 10.5713/ajas.19.0884

**Published:** 2020-05-12

**Authors:** Bin Liu, Linyuan Shen, Zhixian Guo, Mailing Gan, Ying Chen, Runling Yang, Lili Niu, Dongmei Jiang, Zhijun Zhong, Xuewei Li, Shunhua Zhang, Li Zhu

**Affiliations:** 1 College of Animal Science and Technology, Sichuan Agricultural University, Chengdu, Sichuan, 611130, China; 2 Farm Animal Genetic Resources Exploration and Innovation Key Laboratory of Sichuan Province, Sichuan Agricultural University, Chengdu, Sichuan, 611130, China; 3 Sichuan Province General Station of Animal Husbandry, Chengdu 610066, China; 4 Agriculture and Rural Bureau of Mabian Yi Autonomous County, Mabian, 614600, China; 5 Sichuan Academy of Animal Sciences, Chengdu 610066, China

**Keywords:** Single Nucleotide Polymorphism (SNP) Chip, Liangshan Pig, Inbreeding Coefficient, Genetic Distance, Genetic Diversity

## Abstract

**Objective:**

To conserve and utilize the genetic resources of a traditional Chinese indigenous pig breed, Liangshan pig, we assessed the genetic diversity, genetic structure, and genetic distance in this study.

**Methods:**

We used 50K single nucleotide polymorphism (SNP) chip for SNP detection of 139 individuals in the Liangshan Pig Conservation Farm.

**Results:**

The genetically closed conserved population consisted of five overlapping generations, and the total effective content of the population (Ne) was 15. The whole population was divided into five boar families and one non-boar family. Among them, the effective size of each generation subpopulation continuously decreased. However, the proportion of polymorphic markers (P_N_) first decreased and then increased. The average genetic distance of these 139 Liangshan pigs was 0.2823±0.0259, and the average genetic distance of the 14 boars was 0.2723±0.0384. Thus, it can be deduced that the genetic distance changed from generation to generation. In the conserved population, 983 runs of homozygosity (ROH) were detected, and the majority of ROH (80%) were within 100 Mb. The inbreeding coefficient calculated based on ROH showed an average value of 0.026 for the whole population. In addition, the inbreeding coefficient of each generation subpopulation initially increased and then decreased. In the pedigree of the whole conserved population, the error rate of paternal information was more than 11.35% while the maternal information was more than 2.13%.

**Conclusion:**

This molecular study of the population genetic structure of Liangshan pig showed loss of genetic diversity during the closed cross-generation reproduction process. It is necessary to improve the mating plan or introduce new outside blood to ensure long-term preservation of Liangshan pig.

## INTRODUCTION

According to the data (2004) from the Domestic Animal Diversity Information System (DAD-IS) and Food and Agricultural Organization (FAO), China produces one-third of the world’s pig breeds [1]. Yet, the number of indigenous breeds has declined sharply in the past 20 years due to breeding selection for lean meat and high growth rate of foreign pig breeds. Liangshan pig is a small, traditional Chinese breed mainly distributed in the mountain areas of Yi Autonomous Prefecture with an altitude of 1,500 to 2,000 m [2]. It is well known for cold tolerance, crude feeding tolerance, and meat quality [3]. Due to a devastating outbreak of African swine fever in 2019, the number of Liangshan pigs declined. Therefore, it is important to study the genetic diversity and the changes in genetic structure of the Liangshan pig population to evaluate and protect China’s abundant genetic resources.

Due to its low cost in recent years, genome-wide genotyping has become beneficial when studying and researching the genetic information of livestock [4]. The genetic variation in some Chinese pig breeds has been extensively studied using high-density single nucleotide polymorphism (SNP) chips or whole genome sequencing [5]. Although the cost involved in genome-wide sequencing has greatly reduced, it is still relatively expensive. As a result, this hinders the use of genome-wide sequencing for the analysis of large-scale samples. In this study, we use 50K SNP chip to analyze genetic diversity, genetic relationship, population structure, and inbreeding coefficient of Liangshan pigs in the conserved population farm.

## MATERIALS AND METHODS

### Animal care

All animal works were conducted according to the guidelines on the care and use of experimental animals established by the Ministry of Agriculture of China. The Animal Care and Ethics Committee of Sichuan Agricultural University specifically approved this study under Permit No. DKY-S2017 6906.

### Animals

Ear tissues from 139 purebred Liangshan pigs were collected for DNA extraction from the Liangshan Pig Conservation Farm of Leshan, Sichuan province. After pedigree data query, all samples were divided into a total of five generation subpopulations (Supplementary Table S1).

### Single nucleotide polymorphism genotyping

DNA was extracted from the ear tissues by phenol-chloroform extraction method [6], and the quality of DNA was detected by ultraviolet spectrophotometry (NanoDrop, 2000; Thermo Scientific, ShangHai, China) and gel electrophoresis (BIO-RAD & DYPC-31BN, Newbio Gi-1, WuHan, China). The qualified 139 DNA samples were genotyped using “Zhongxin-I” Porcine Breeding Chip (Beijing Compass Agritechnology Co., Ltd., Beijing, China), which contains 51,315 SNPs. Quality control of genotype data was performed using PLINK (v1.90) software [7]. Only autosomal loci were used; SNPs with a minor allele frequency less than 0.05 and with a call rate less than 90% were eliminated.

### Analysis of genetic diversity

Effective population size (Ne) refers to the size of an ideal population with the same gene frequency variance or the same inbreeding coefficient increment (hybridity attenuation rate) as the actual population, which is estimated based on the level of linkage disequilibrium [8]. We used SNeP (v1.1) software to calculate Ne [8].

Polymorphic marker ratio (P_N_) refers to the proportion of polymorphic loci in the target population to the total number of loci. We first calculated the minimum allele frequency for each locus using PLINK (v1.90) [7] and then calculated P_N_ using a self-programmed *R* script [9]. We calculated P_N_ using the formula as follows:

$$P_{N}=\frac{M}{N}$$

where *M* is the number of sites that exhibit polymorphism and *N* is the total number of sites.

Expected heterozygosity (He) refers to the probability of heterozygosity at any one of the individuals in the population; observed heterozygosity (Ho) refers to the ratio of the number of individuals in a population where a locus is heterozygous to the total number of individuals. When the Ho is less than the He, we speculate that the population has experienced selection or inbreeding; if the Ho is more than the He, the population may have introduced some other varieties. We used PLINK (v1.90) to calculate He and Ho [7].

### Calculation of genetic distance and genetic relationship

We used PLINK (v1.90) to calculate idengtical by state (IBS) distances and *R* script to build heat maps. IBS refers to the DNA fragment identical by descent in two or more individuals, and these DNA fragments have the same base sequence. IBS only considers the similarity of genetic markers or alleles between individuals, regardless of whether they come from the same ancestor or not. Therefore, there is no need for parental genotyping. The genetic distance based on IBS can still analyze the genetic relationship of the population without information on the pedigree or ancestral samples. We used ***G*** matrix (v2) and *R* to calculate kinship values and heat maps [10]. ***G*** matrix is a genomic relationship matrix constructed with whole genome markers. Since the pedigree information of a conserved population is usually not recorded, ***G*** matrix is suitable for calculating the genetic relationship.

### Analysis of population structure

After quality control of the genotype data, 36,592 SNP loci were used to analyze the population structure. The population structure is mainly clustered by the neighboring method (neighbor-joining [NJ]) and based on IBS distance matrix [11]. Based on the analysis, we can roughly infer which Liangshan pig individuals are close in blood in general, samples originated from the same family group. We used PLINK (v1.90) for population structure analysis [7].

### Inbreeding coefficient analysis

Widespread in all populations, runs of homozygosity (ROH) are contiguous segments in an individual genome due to complete transfer of a homologous haplotype from a parent to a progeny. The length and frequency of ROH can reflect the group history. A long ROH indicates recent inbreeding, while a short ROH indicates ancient inbreeding. First, the length of ROH in each sample was calculated by PLINK (v1.90) [7]. Then the ratio of the total length of the ROH fragment to the total length of autosomal genome was calculated to get the coefficient of inbreeding based on ROH [12] using the following formula:

$$F_{ROH}\frac{\sum_{k}Length({ROH}_{k})}{L}$$

where *k* is the number of ROH in the individual and *L* is the length of the autosomal genome of the species (porcine v10.2 version of the genome, autosomal length is approximately 2,450,713 Kb).

### Pedigree accuracy analysis

Pedigree accuracy was analyzed using Mendelian errors based on paired individuals (individual-parent, individual-mother) with genotypes in the pedigree [13]. Mendelian error indicates which of an individual’s allele is not from any of its biological parents, and the Mendelian error rate identifies the proportion of markers that have made a mistake in the calculation. If a pair of individuals with a Mendel error rate greater than 1% is discovered in the pedigree, then the pedigree is considered wrong and will be found in other homosexual samples that provide genotypes. If a sample with a Mendel error rate below 1% is not found, the individual is considered missing. If multiple samples with a Mendel error rate below 1% are found, the sample with the smallest Mendel error rate (born earlier to offspring) is selected as the true parent of the individual.

## RESULTS

### Single nucleotide polymorphism characteristics

A total of 51,315 SNP loci were detected from 139 samples. PLINK (v1.90) [7] was used to remove loci on sex chromosomes, and SNP genotyping data of 44,739 SNP loci were used for the subsequent analysis.

### Pedigree accuracy of the conserved population of Liangshan pigs

Information on a total of 26 sires and 19 dams were available in the pedigree record of 139 Liangshan pigs (Table 1). After genotypic analysis, 4 wrong sire records were corrected. We found that the information on 12 sires and 3 dams were erroneous; however, their true parents were not detected via genotyping. A total of 97 sires and 117 dams were excluded from the experimental group tested by the chip. Therefore, it was impossible to judge the accuracy of the information. Altogether, in the conserved population, the pedigree error rate of sire information was more than 11.51%, and the error rate of dam information was more than 2.16%.

### Genetic diversity of the Liangshan pigs

The Ne of these 139 purebred pigs of Liangshan Pig Conservation Farm was 15.00 and the proportion of P_N_ was 0.8393. We observed significant differences in population effective content (Ne) among the different generations. The sample size of the F5 generation subpopulation was 10 and the effective population content was 2.31. The sample size of the F3 generation subpopulation was 41 and the effective population content was 7.89. Lowest proportion of P_N_ (0.8060) was in the F4 generation subpopulation and highest (0.8393) was in the F5 generation subpopulation. In the conserved population and all these five generations, the He (0.3478) was less than the Ho (0.3551), which suggests some other species mixed in the population (Table 2).

### Genetic distance of the conserved population of Liangshan pigs

**Figure 1:**
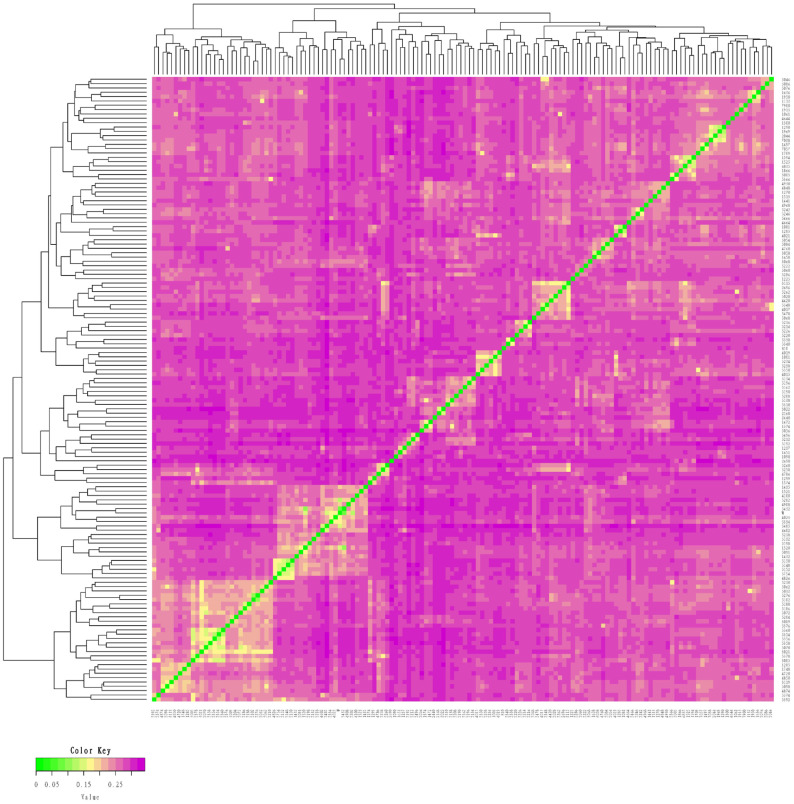
Identity by state (IBS) distance matrix of the conserved population of Liangshan pigs. Using Plink software and R package to make IBS heat map. Each small square in IBS distance matrix represents the genetic distance value between two pairs from the first sample to the last sample. The larger the value is, the closer it is to red, that is, the larger the genetic distance between two individuals is, that is, the two individuals are not similar, and vice versa.

**Figure 2:**
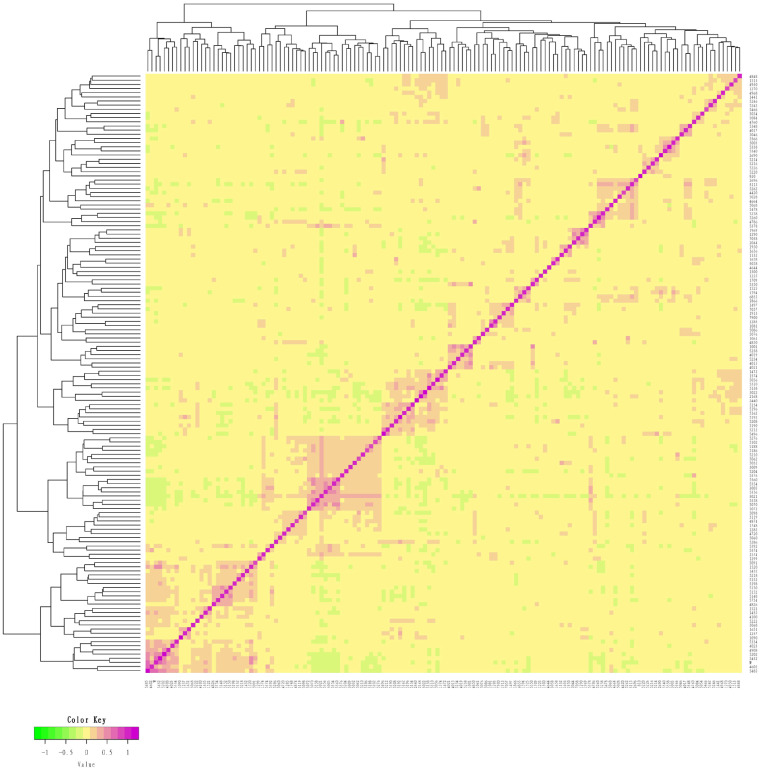
*G* matrix of the conserved population of Liangshan pigs. Using *G* matrix software and R package to make *G* matrix heat map. Each small square represents the value of the relationship between two pairs from the first sample to the last sample. The larger the value is, the closer it is to red, that is, the closer the relationship between two individuals is.

We measured an IBS distance that varied from 0.1261 to 0.3442 for the conserved population of Liangshan pigs and the generation subpopulations. We also determined that the overall population average genetic distance to be 0.2823±0.0259. It showed that the average genetic distance among Liangshan pigs is far apart, and the variation of the population is quite large. The genetic distance among 14 breeding boars ranged from 0.1659 to 0.3136, and the average genetic distance was 0.2723±0.0384. The genetic distances of all the five generation subpopulations ranged between 0.2765 and 0.2882. The genetic distance of the F5 generation subpopulation was the lowest (0.2765±0.0253) and the F4 generation subpopulation was the highest (0.2882±0.0279) (Table 3). Visualization results of IBS distance matrix and ***G*** matrix of the conserved population and the different generation subpopulations showed that most of the individuals have moderate genetic relationship and some have high genetic relationship. These findings indicated a potential inbreeding trend among these individuals (Figures 1, 2), and therefore, we need to pay special attention to their mating plan.

### Population family genetic structure of Liangshan pigs

**Figure 3:**
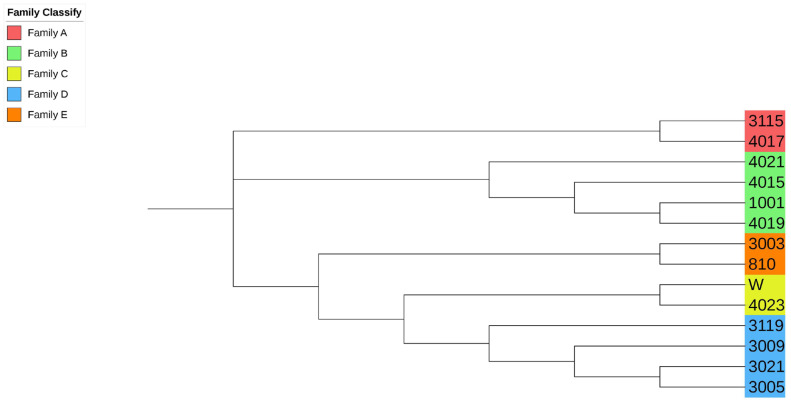
Evolutionary tree of boar samples in the conserved population. Samples labeled with the same color are evaluated as the same family. All boars include 5 families.

**Figure 4:**
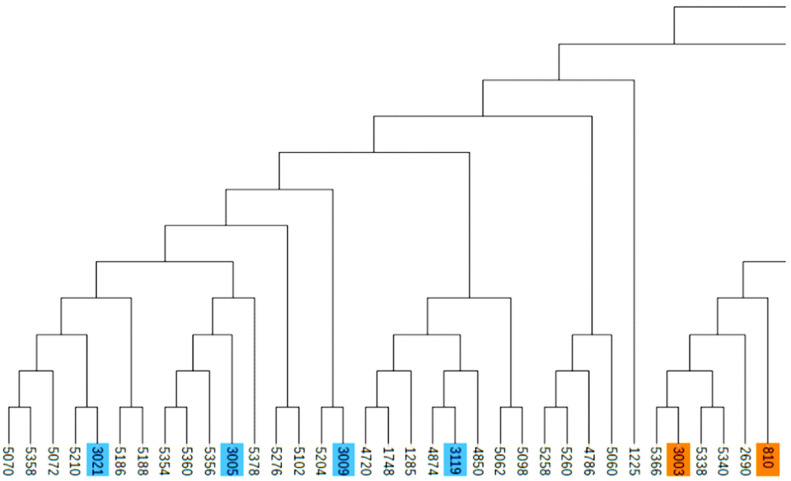
Partial screenshot of the phylogenetic tree. Samples labeled with the same color are evaluated as the same family.

Due to the importance of boars in the conservation process, this study combined the ***G*** matrix and IBS distance matrix to analyze the phylogenetic tree of all 14 boars in the population using the NJ method. The existing boars were divided into five different consanguinity families. Samples labeled with the same color in the evolutionary tree were evaluated as the same family, and the same consanguinity family branches were divided into the second or more inner clustering units in the evolutionary tree (Figure 3). Based on NJ tree and the relationship matrix, combined with the relationship between sows and boars of different families, all individuals in the conserved population were eventually divided into five large families. In addition to the five families, 49 sows were genetically less related to any of the tested breeding boars (genetic relationship coefficient less than 0.1). Therefore, they were classified as another family (Table 4, Figure 4).

### Inbreeding coefficient of the Liangshan pigs based on runs of homozygosity

**Figure 5:**
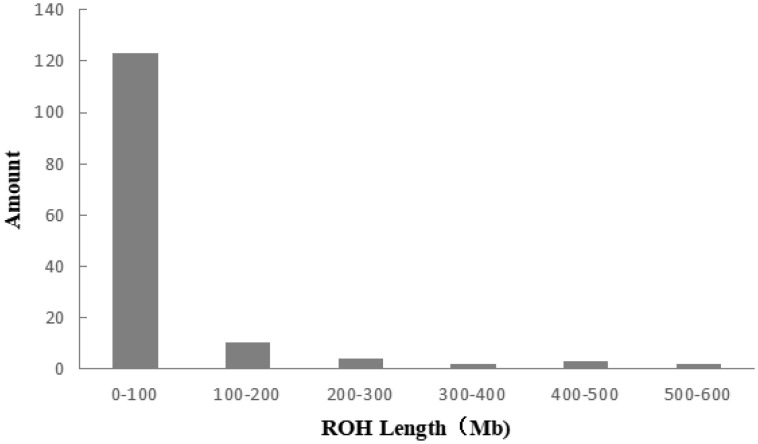
Distribution of ROH lengths in the individuals of Liangshan pigs. Taking 50 SNPs as windows, moving 5 SNPs at a time and calculating the length of ROH. The length of ROH in 0 to 100 Mb is the largest in Liangshan pigs. ROH, runs of homozygosity; SNP, single nucleotide polymorphism.

**Figure 6:**
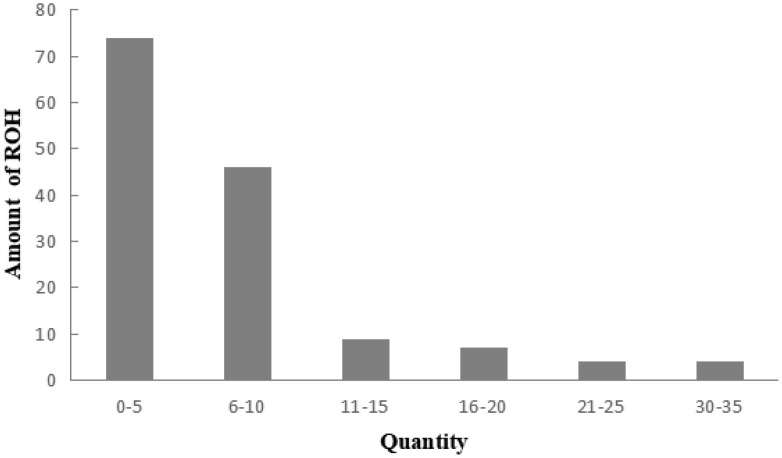
Distribution of runs of homozygosity (ROH) in the individuals of Liangshan pigs.

**Figure 7:**
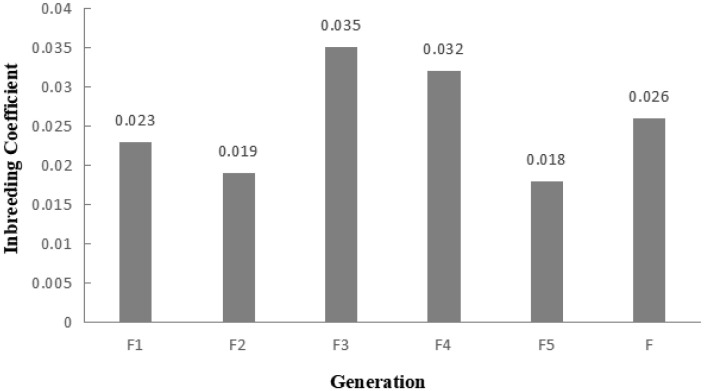
Average inbreeding coefficient of the conserved population and different generation subpopulations. By calculating the proportion of the total length of runs of homozygosity (ROH) fragment to the total length of autosomal genome, the coefficient of inbreeding based on ROH was obtained.

A total of 983 ROH were detected in these 139 Liangshan pigs. The total length of ROH of each individual ranged between 2.76 Mb and 522.51 Mb, and the average ROH length of the conserved population was 63.24±101.92 Mb. Liangshan pig with ROH less than 100 Mb has the largest number of individuals (86.11% of the protected population; Figure 5) The total number of ROH in each Liangshan pig varied between 0 and 33 (an average of 7.07±6.48), and the individuals with 0 to 5 ROH was the largest in the conserved population (Figure 6). Overall, the number of ROH positively correlated with chromosome length. We obtained the inbreeding coefficient value of each individual through ROH statistics. In the genetically closed conserved population of Liangshan pigs, the average inbreeding coefficient of the conserved population was 0.026. However, the average inbreeding coefficient of each generation subpopulation changed significantly. Among the different generation subpopulations, the highest inbreeding coefficient was 0.035 in the third generation and the lowest was 0.018 in the fifth generation (Figure 7).

## DISCUSSION

### Pedigree accuracy analysis

In the breeding work, data recording errors or defects are inevitable, which also decrease the accuracy and integrity of pedigrees. In indigenous pig conservation farms, the working conditions are normally very tough. So far no database has been established and genetic information relies on the handwriting in the records. It is impossible to exclude errors in genealogical records caused by the negligence of staff members or existing unrecorded genealogical information. According to statistics, the average pedigree error rate in animal and tree breeding population can reach up to 10% [14,15]. The genome homozygosity index based on whole genomic markers can reflect the inbreeding degree of individuals. It has more application in revealing the actual inbreeding level and inbreeding genetic effect of the population [16]. In the absence of pedigree data, the correction of pedigree inbreeding coefficient with genome inbreeding coefficient is a more accurate tool to measure individual inbreeding degree [17]. Correcting pedigree errors can improve best linear unbiased prediction-breeding value predictions and improve heritability estimates [16], which can effectively protect the Liangshan pig population. Through our analysis, we discovered an error rate greater than 11.35% in the paternal information and more than 2.13% in maternal information on Liangshan pigs, thus meaning that the error rate in paternal information was more than that in maternal information. This may be because the maternal information normally comes from daily farm records on reproduction and mating; however, there are many times in the process of artificial insemination. This may further lead to errors in paternal information. Therefore, we need to strengthen the accuracy of pedigree records in the subsequent breeding works on Liangshan pigs.

### Genetic diversity

Using genotype data to assess the Ne of the current population is a very important research hotspot in conservation genetics [18,19]. High-density SNP panels improve the accuracy in population parameter estimation, including Ne [20]. Our analysis revealed that the conserved population of Liangshan pigs has remained genetically closed and kept reproducing from the formation of the basic group till the fifth generation. Now, the whole conserved population consists of five overlapping generations. We recorded an Ne of 15 (the whole conservation population), and with increasing generations, the Ne of each generation subpopulation showed a significant variation. The Ne of the fifth generation was only 2.31, which may be because only 10 samples were available in this generation. Using the same method, Shin et al [21] estimated the effective population content of a Landrace pig population (1,128 individuals) as 92.27. Due to the small size of the population and their unfavorable natural habitat (mountain areas), Liangshan pigs has experienced an extinction crisis. By 2010, the conserved population was established, but the Ne of Liangshan pigs still remains low after five generations of closed reproduction due to limited independent blood families of the basic group. This is currently a major problem that the vast majority of indigenous livestock and poultry breed in China face. Therefore, in the conservation of indigenous pig breeds, we need to actively introduce new consanguinity, especially new boar consanguinity, and pay special attention to selection and mating schemes to prevent the loss of independent consanguinity in the alternation of generations. Comprehensive genetic diversity analysis showed that the SNP polymorphism (P_N_) kept varying among different gene ratio subpopulations. The SNP polymorphism (P_N_) of the whole conserved population was 0.8393, which was less than that of other Chinese indigenous breeds (Bihu, Chalu, Chun’an, Jinghua, Shengxianhua, Lanxihua, and Jiaxinghei) [22]. The Ho was more than the He for all the generation subpopulations and for the whole conserved population, which indicates that the conserved population contained mixed-breeds and need to be further purified by eliminating impure individuals. In the conserved population of Liangshan pigs, the genetic diversity decreased after generations of closed reproduction. In addition, the heterozygosity of the population (both He and Ho) was low, which may be due to inevitable inbreeding or loss of less productive families. This is also common in other small-sized Chinese indigenous livestock and poultry breeds [14].

### Genetic distance of the conserved population and the generation subpopulations

In order to protect the population from the influence of exotic varieties, most of the conserved livestock populations in China remain genetically closed. Therefore, it is important to study genetic distance, genetic relationship, and genetic structure of the conserved population, especially the multi-generation overlapping population, to maintain sustainable development [23]. IBS showed that the genetic distance of the whole conserved population and of the 14 breeding boars was 0.2823 and 0.2723, respectively. After five generations of closed reproduction, the genetic distance of the subpopulations slightly changed, and the fifth generation subpopulation had the lowest genetic distance (0.2765). These findings indicate that the conserved population experienced a selection pressure during generation alternation, which changed the genetic structure of the closed conserved population.

### Analysis of population genetic structure

In order to analyze the degree of genetic background difference of the whole population, we used the NJ method and clustered the samples based on IBS distance matrix [5]. In the NJ tree, the whole conserved population was divided into five different families with boars and a family without boars. At present, the number and inbreeding degree of boars from each family are not balanced in the whole population. In three families, there were only two boars. Therefore, in the subsequent conservation process, we need to pay special attention to the number of pigs in each family during mating plans to avoid the loss of blood lines and maintain a balanced family structure. After identifying the effective blood relationship of breeding boars by SNP chip, we can use this as a guide to select for reproduction and to construct frozen semen banks of different breeds. This will help reduce the cost of genetic resources and improve the efficiency of conservation.

### Analysis of inbreeding coefficient based on runs of homozygosity

Chromosome fragments containing homozygous SNP genotypes are used to infer possible haploids inherited by the same individual, and ROH (Froh) is used to estimate the inbreeding coefficient of the genome [24]. In addition, a long ROH indicates a more recent genetic relationship, a short ROH denotes ancient inbreeding, and a greater quantity of ROH fragments implies a higher possibility of inbreeding in the family [25]. At present, the number of individuals with ROH length within 100 MB is the largest in the whole population, and especially the number of ROH within 5 is the largest. This may be due to the different generations and selection strategies used by the conserved farm, as well as the different degrees of selection pressure on the traits of these lines [26]. Bosse et al [27] found that ROH larger than 5 Mb could be detected using a 60K SNP chip, and ROH larger than 5 Mb were as accurate as the whole genome sequencing. In this experiment, we determined 0 Mb to be the shortest ROH of Liangshan pigs and 63.24 Mb as the average length, which proves the accuracy and validity of our results. In addition, our results showed that 86.11% of ROH were shorter than 100 Mb, which suggests ancient inbreeding. Due to the limited population size of the indigenous pig breeds and the relatively closed operation system, after reproducing for successive generations and the aggravation of overlapping generations, the inbreeding coefficient of the population kept increasing and the genetic structure of the conserved population changed dramatically. Our analysis revealed that the inbreeding coefficient of the genetically closed conserved population of Liangshan pigs kept increasing, and the inbreeding coefficient of the third generation subpopulation was the highest (3.5%). Due to the introduction of certain boars after that, the inbreeding coefficient decreased. Now, the inbreeding coefficient of the whole conserved population remains at 2.6%.

## CONCLUSION

By studying the population genetic structure of Liangshan pigs at the molecular level, we found that the effective population content and the genetic diversity of the conserved population were low during the process of subculture, which led to a change in the genetic structure of the population to a certain extent. This variation in genetic structure may be caused by other breeds that got mixed in the population. To protect LiangShan pigs, it is necessary to further purify the population and increase the matching strength or import foreign consanguinity to ensure long-term preservation of genetic resources. Further study on the genetic structure of the local protected population in China at the molecular level will effectively help to protect these excellent local varieties.

## Supplementary Information



## Figures and Tables

**Table 1:** Rectifying results of Liangshan pig pedigree

Result type	Number of sires	Number of dams
No genotype was provided.	97	117
The original pedigree did not match and the real parents were not detected.	12	3
The original pedigree did not match and the real parents were detected.	4	0
Matching with the original pedigree	26	19

**Table 2:** Genetic diversity parameters of Liangshan pigs by generations

Generation	Ne	P_N_	He	Ho
F1	6.70	0.8339	0.3526	0.3677
F2	7.78	0.8339	0.3477	0.3604
F3	7.89	0.8146	0.3507	0.3583
F4	4.88	0.8060	0.3563	0.3697
F5	2.31	0.8393	0.3327	0.3595
F	15.00	0.8393	0.3478	0.3551

Ne, effective population content; P_N_, the proportion of single nucleotide polymorphisms (SNPs) that displayed polymorphism in 44,739 SNPs selected from the 50K panel; He, expected heterozygosity; Ho, observed heterozygosity.

**Table 3:** Identity by state genetic distance of Liangshan pigs

Generation	Max	Min	Average	Standard deviation
F1	0.3442	01372	0.2826	0.0264
F2	0.3248	0.1565	02797	0.0218
F3	0.3363	0.1261	0.2822	0.0271
F4	0.3395	0.1409	0.2882	0.0279
F5	0.3052	0.1953	0.2765	0.0253
F	0.3442	0.1261	0.2823	0.0259
Boar	0.3136	0.1659	0.2723	0.0384

Max, maximum value; Min, minimum value.

**Table 4:** Consanguinity family construction in the conserved population

Family	Gender	Quantity
Family A	Boar	2
	Sow	17
Family B	Boar	4
	Sow	11
Family C	Boar	2
	Sow	21
Family D	Boar	4
	Sow	32
Family E	Boar	2
	Sow	8
Other	Sow	49
