# Supplementary material for: Single nucleotide polymorphism-based analysis of the genetic structure of Liangshan pig population
Source: Anim Biosci. 2020 May 12;34(7):1105–15. doi: 10.5713/ajas.19.0884 (PMC8255872; doi:10.5713/ajas.19.0884)
Supplement: Supplementary file 1 [file ajas-19-0884-suppl.pdf]

Supplementaty table：

Table S1 Liangshan pig distribution by generations

Generation	Boar	Sow	Total
F1	4	28	32
F2	2	31	33
F3	6	35	41
F4	1	22	23
F5	1	9	10
Total	14	125	139
